# 3-year randomized clinical trial to evaluate the performance of posterior composite restorations lined with ion-releasing materials

**DOI:** 10.1038/s41598-024-55329-6

**Published:** 2024-02-28

**Authors:** Basma Ahmed, Ramy Ahmed Wafaie, Hamdi H. Hamama, Salah Hasab Mahmoud

**Affiliations:** 1 Conservative Dentistry Department, Faculty of Oral and Dental Medicine, Delta University for Science and Technology, Gamasa, Egypt; 2 Conservative Dentistry Department, Faculty of Dentistry, Mansoura University, Mansoura, 35516 Egypt; 3 Faculty of Dentistry, New-Mansoura University, New-Mansoura, Egypt

**Keywords:** Deep caries, Clinical trial, Cavity liners, Ion-releasing, Selective caries removal, Dental materials, Dentistry, Restorative dentistry, Bonded restorations

## Abstract

To evaluate the impact of using ion-releasing liners on the 3-year clinical performance of posterior resin composite restorations after selective caries excavation with polymer burs. 20 patients were enrolled in this trial. Each patient had two deep carious lesions, one on each side of the mouth. After selective caries removal using polymer bur (PolyBur P1, Komet, Brasseler GmbH Co. KG, Lemgo, Germany), cavities were lined with bioactive ionic resin composite (Activa Bioactive Base/Liner, Pulpdent, Watertown, MA, USA) or resin-modified glass ionomer liner (Riva Light Cure, SDI, Bayswater, Victoria, Australia). All cavities were then restored with nanofilled resin composite (Filtek Z350XT, 3M Oral Care, St. Paul, MN, USA). All the tested materials were placed according to the manufacturers’ instructions. Clinical evaluation was accomplished using World Dental Federation (FDI) criteria at baseline and after 6 months, 1, 2, and 3 years. Data were analyzed using Mann–whitney U and Friedman tests (p < 0.05). The success rates were 100% for all resin composite restorations either lined with ion-releasing resin composite or resin-modified glass ionomer liner. Mann–whitney U test revealed that there were no statistically significant differences between both ion-releasing lining material groups for all criteria during the follow-up periods (p > 0.05). Resin composite restorations showed acceptable clinical performance over 3 years either lined with bioactive ionic or resin-modified glass ionomer liners after selective caries excavation preserving pulp vitality. After the 3-year follow-up period, Activa Bioactive and Riva Light Cure liners were clinically effective and they exhibited with the overlying composite restorations successful clinical performance.

**Trial registration number:** NCT05470959. Date of registration: 22/7/2022. Retrospectively registered.

## Introduction

Dental caries remains the most prevailing global disease that has a deleterious effect on the oral health among adults^1–3^. Managing deep carious lesions still represents a great challenge in determining the best treatment option that provides optimum prognosis through retaining pulp vitality and preventing apical lesions^4^. The traditional management was based upon non-selective caries removal to hard dentin strategy including complete removal of all infected and affected dentin to preclude further cariogenic activity offering well-mineralized dentin^5^. However, complete caries excavation frequently results in pulp exposures and weakening of tooth structure which compromises the success of dental treatment^6^. Therefore, less invasive alternative approaches have been adopted. One of these approaches is selective caries excavation which aims to remove the outer infected dentin layer, while maintaining the deeper remineralizeable affected layer^7^. A previously conducted systematic review^3^ confirmed that selective caries removal was an effective method for conservative excavation of deep carious lesions in permanent teeth.

The rapid development of the concurrent cariology concepts and the advances in dental materials have brought new techniques to the dental field in order to minimize the unnecessary sacrifice of tooth substrate^8^. Self-limiting polymer burs were released to dental market with slightly inferior mechanical properties than sound dentin. Their blades were designed to remove infected dentin by locally depressing the soft carious tissue and pushing it forward along the surface until it ruptures and carried out of the cavity^9,10^. When these burs touch sound or caries-affected dentin, they become dull and produce vibrations making further cutting impossible^11^. Toledano et al.^12^ reported that polymer burs showed higher preservation of affected dentin after deep caries excavation when compared to carbide burs. Another in-vitro study^13^ confirmed that polymer burs were as effective as tungsten carbide burs in dentin caries excavation.

Pulp protection using cavity liners is a crucial step in managing deep carious lesions. These intermediary liners exhibit several advantages such as antibacterial property, remineralization potential, thermal/electric insulation, and they act as chemical barriers protecting the pulp^14^. Qureshi et al.^15^ reported that the ideal cavity lining material should promote reparative dentin formation, preserve pulp vitality, release fluoride, attach to restorative materials, and inhibit bacterial leakage. Previously, calcium hydroxide was the most commonly used liner during step-wise excavation method^14^. However, a systematic review^16^ reported failure of calcium hydroxide lining material in incompletely excavated teeth due to over-time degradation, inferior mechanical properties, and difficulties in dentin barrier formation^17^. Nowadays, several promising ion-releasing lining materials have replaced calcium hydroxide such as resin-modified glass ionomer liners (RMGI)^18^. RMGIs were introduced as an attempt to overcome the drawbacks of their conventional predecessors, while maintaining the clinical advantages of fluoride release and antibacterial activity. Moreover, they offer enhanced physical properties, controllable working time, less moisture sensitivity, and stable bonding to tooth structure due to micromechanical adhesion^19^.

A new lining material called Activa Bioactive was launched in 2014. The manufacturer claims that this material exhibits improved strength, esthetics and good physical properties. Also, it show high ability for calcium, phosphate, and fluoride release which stimulates the remineralization and apatite formation as claimed by the manufacturer^18^. These intermediatory materials showed similarity with natural dentin from physical and chemical aspects. This similarity is attributed to their ionic resin matrix, shock-absorbing resinous component, as well as, bioactive filler ingredient^20^. The manufacturer also claimed that this material does not require additional pretreatment before application due to its self-adhesive property and chemical bonding to tooth structure. A 1-year follow-up randomized clinical trial reported that Activa Bioactive liner showed high clinical and radiographic success rates and could be used safely in indirect pulp treatment^21^.

To date and according to the authors’ knowledge, Activa Bioactive liner has scant literature comparing its effect to other lining materials in deep cavities after selective caries excavation. Moreover, no randomized clinical trials evaluating the long-term clinical success of posterior resin composite restorations lined with this new material using all the FDI criteria were found. The authors believe that RMGI lining material could be also considered as an ion-releasing intermediary material. Hence, this clinical study was designed to comprehensively evaluate the impact of using ion-releasing liners on the 3-year clinical performance of posterior resin composite restorations after selective caries excavation with polymer burs using the FDI evaluation criteria. The primary clinical outcome was the clinical success rate of composite restorations after 3 years, while the esthetic, functional, and biological clinical criteria were also evaluated as secondary outcomes. The null hypothesis tested was that there would be no significant difference in the clinical outcome of posterior resin composite restorations lined with Activa Bioactive or Riva Light Cure liners.

## Materials and methods

### Ethical approval and trial registration

This clinical trial was approved by the Dental Research Ethics Committee of Mansoura University (approval no. M19091019). The selected volunteers were participated in this trial after signing a consent which guaranteed their confidentiality and informed them about steps, duration, place, benefits, and possible side effects of the research. This trial was registered at https://www.clinicaltrials.gov under a registration number (NCT05470959).

### Trial design and blinding

**Figure 1:**
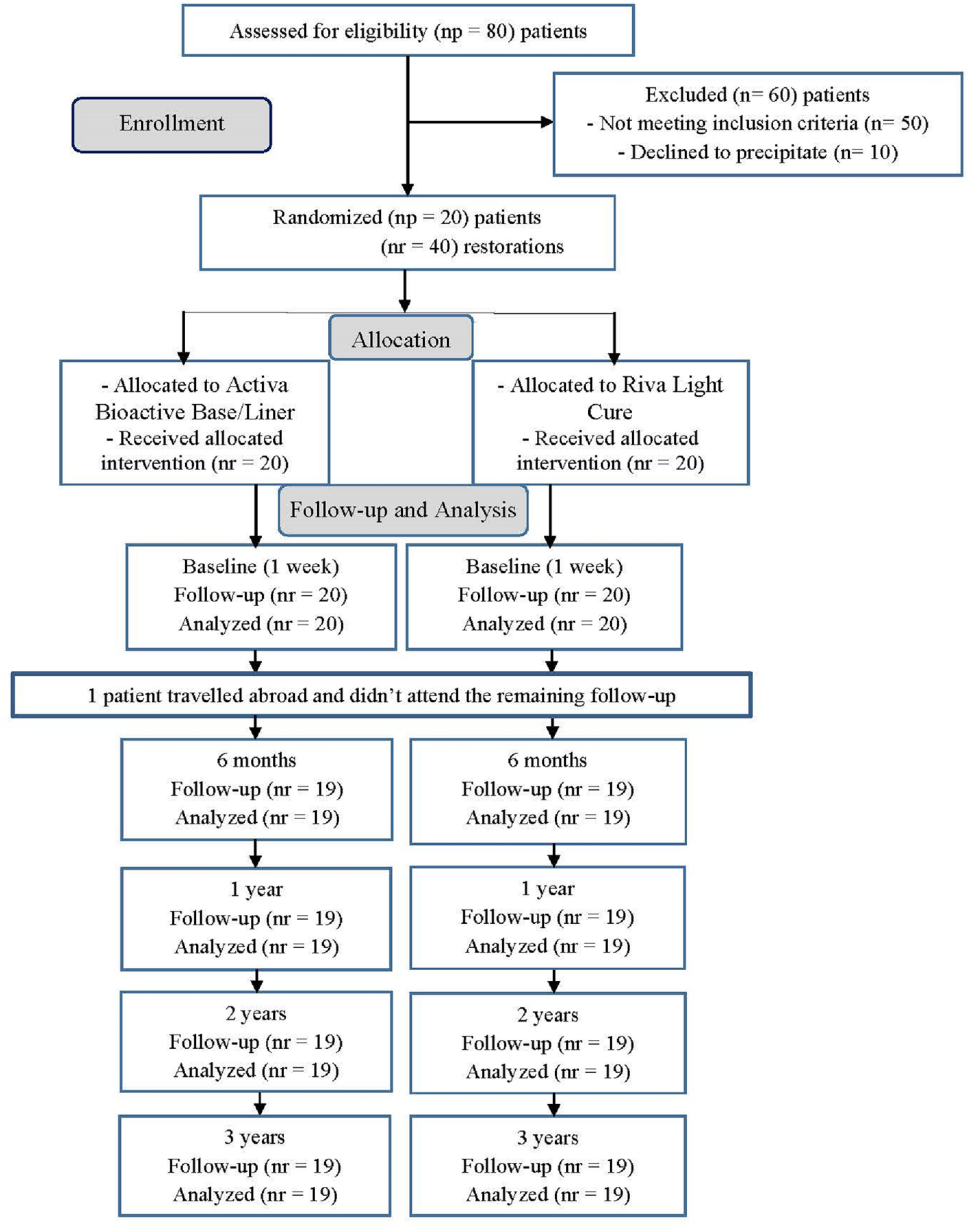
A CONSORT flow diagram showing the number of enrolled patients and evaluated restorations during this study.

This was a randomized clinical trial along with split mouth design. It was executed following guidelines and recommendations of World Dental Federation (FDI)^22,23^ and Consolidated Standards of Reporting Trials (CONSORT)^24^. Neither the patients nor the evaluators knew which lining material was used, thus resulting in a double-blind study (Fig. 1).

### Sample size calculation

The primary outcome of this cohort RCT was the clinical success rate of composite restorations after 3 years, considering all properties evaluated. The sample size was calculated on the basis of the clinical success rate of posterior composite restorations observed in a previous study^14^, testing resin-modified glass ionomer liner after selective/partial caries removal with a success rate of 97% after 12 months using statistical software program (G*Power, Ver.3.1.9.1, Dusseldorf, Germany) at 95% confidence level with a statistical power of 80% and a significance level (alpha value = 5%). Therefore, a representative sample composed of 20 teeth had to be included in each treatment group taking into consideration the possible patient dropout during the trial period.

### Randomization

Each selected patient received dental treatment for deep carious lesions using two different pulp lining material. Each material was placed in a different side of mouth randomly as determined by flipping a coin^25^. A staff member not involved in the clinical trial performed the randomization process and the allocation assignment was revealed only on the day of the restorative procedure in order to avoid the bias.

### Recruitment and eligibility criteria

20 patients were selected from regular attendants of Operative Dental Clinic at the Faculty of Dentistry, Mansoura University, Egypt. The average age of patients involved in this study was 25 years (range, 20–35 years).

Inclusion criteria were as follows: a patient presenting with (1) a need for at least two deep carious lesions (ICDAS 5 or 6) one on each side of the mouth, (2) symptomless and vital teeth with no signs for pulpal inflammation or pathological lesions, (3) good oral hygiene, and (4) good likelihood of recall availability. Exclusion criteria were as follows: (1) adverse medical history, allergies or systemic diseases, (2) pregnant or lactating females, (3) visibly cracked teeth, (4) sensitivity to percussion, and (5) severe or chronic periodontal problems^14,26^.

### Clinical procedures

The lining materials used in this study were: Bioactive ionic resin composite; Activa Bioactive (Pulpdent, Watertown, MA, USA) and Riva Light Cure (SDI, Bayswater, Victoria, Australia). A nanofilled resin composite; Filtek Z350XT (3M Oral Care, St. Paul, MN, USA) was used as a final restoration. The materials used in this clinical study are listed in Table 1. All these materials were used in accordance with the manufacturers’ instructions. One experienced dentist performed the clinical procedures in 20 patients (11 male and 9 female) fulfilling the inclusion and exclusion criteria.

**Table 1:** Materials used in the study.

Materials	Type	Manufacture	Composition	Batch no.
Filtek Z350 XT	Nanofilled Resin Composite	3M Oral Care	Matrix: Bis-GMA, UDMA, TEGDMA, PEGDMA, Bis-EMA Filler: Combination of non-agglomerated/non-aggregated 20 nm silica filler, non- agglomerated/non-aggregated 4 to 11 nm zirconia filler, and aggregated zirconia/silica cluster filler	NC93014
Single bond Universal	Universal Adhesive	3M Oral Care	MDP phosphate monomer, dimethacrylate resin, HEMA, filler, ethanol, water, initiators, silane, vitrebond copolymer	517571
Scotchbond Universal Etchant	Etching Gel	3M Oral Care	Phosphoric acid 35%, water and synthetic amorphous silica	603087
Activa Bioactive Base/Liner	Bioactive Ionic Resin with Reactive Glass Filler	Pulpdent	Blend of diurethane and other methacrylates with modified polyacrylic acid, amorphous silica, sodium fluoride.	181213
Riva conditioner	Polyacrylic Acid Conditioner	SDI Limited	Polyacrylic acid 25–30% by wt	200250
Riva Light Cure	Resin-Modified Glass Ionomer	SDI Limited	2-hydroxyethyl methacrylate, acrylic acid homopolymer, dimethacrylate cross-linker, acidic monomer, tartaric acid, glass powder.	J2102033

Preoperative digital photographs were taken as part of dental screening. To detect the depth of the carious lesions, bitewing radiographs were examined using the common classification system that consists of E0 (no lesion), E1 (lesion within the outer half of enamel), E2 (lesion within the inner half of enamel), D1 (lesion within the outer third of dentin), D2 (lesion within the middle third of dentin), and D3 (lesion within the inner third of dentin)^27,28^. After assessing all radiographs under optimal light conditions, the depth of proximal carious lesions in all cases was D3. Pulp vitality was confirmed by application of cold dry ice^29^. Dental plaque and salivary pellicle were removed from teeth surface by using pumice-water slurry and rubber cup^14^.

After receiving local anesthesia and rubber dam application, cavity preparation started by removing carious tissue using diamond round and straight fissure instruments (801.314.012 & 835KR. 314.014, Komet, Brasseler, Lemgo, Germany) at high-speed handpiece (Sirona T3, Bensheim, Germany) under copious air–water cooling. Deep carious lesions on pulpal and axial walls were managed by selective caries excavation using polymer bur PolyBur P1 (Komet, Brasseler GmbH Co. KG, Lemgo, Germany) at low-speed hand-piece with circular movements starting from the center of the lesion to the periphery. The caries excavation stopped when polymer burs reached caries-affected dentin as they became abraded or blunted with no further ability to remove tissue^30^. The cavity depth was evaluated using Prepometer (Hager & Werken, Duisburg, Germany). The prepared cavities were then finished using extra-fine diamond instrument (835 KREF.314.012, Komet) in order to round all line angles.

After cavity preparation, each patient received both lining material; Activa Bioactive and Riva Light Cure. These materials were placed only over the pulpal portion of preparations. Regarding Activa Bioactive, the material was applied directly to pulpal floor without placement of any additional pretreatment as recommended by the manufacturer, and then light cured for 20 s by using a light emitting diode (LED) curing unit Elipar S10 (3M Oral Care, St. Paul, MN, USA) which had a wave length between 430 and 480 nm and a light intensity 1200 mW/cm^2^. For cavities receiving Riva Light Cure, dentin was conditioned with 25–30% polyacrylic acid (Riva Conditioner, SDI Bayswater, Victoria, Australia) for 10 s, then rinsed thoroughly with water and gently dried with oil-free air without desiccating the surface keeping dentin moist. Riva Light Cure was then applied and light cured for 20 s.

**Figure 2:**
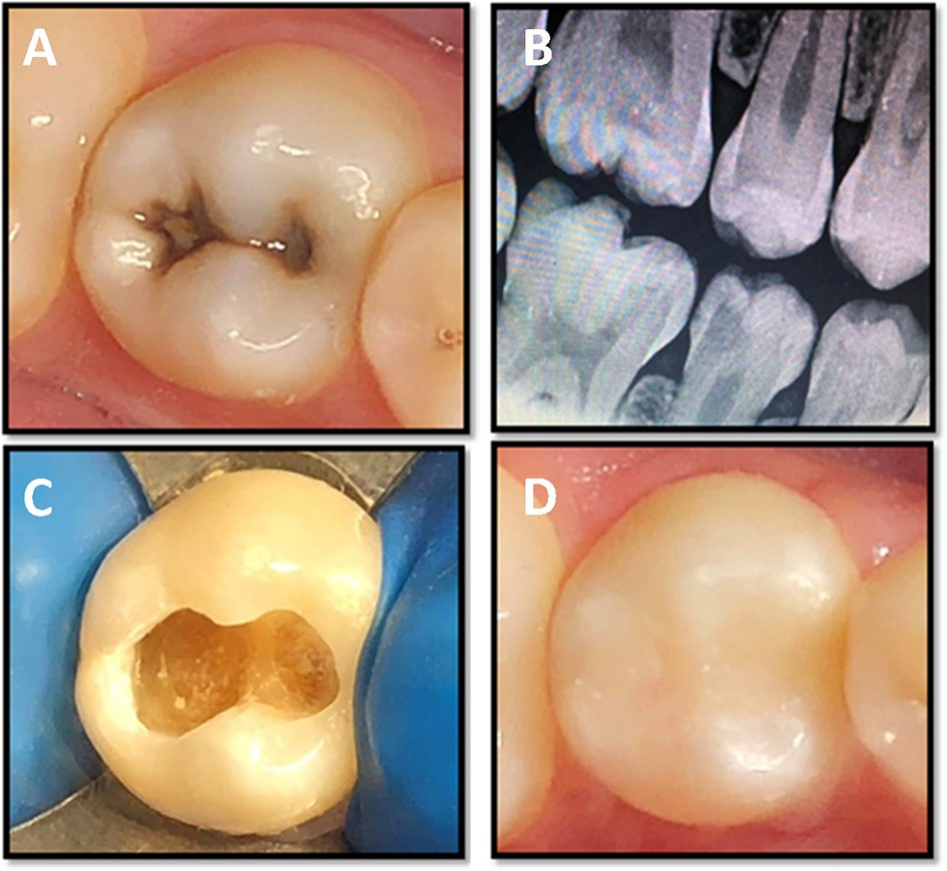
A representative of mandibular second premolar tooth showing; (**A**) preoperative photograph, (**B**) preoperative digital bitewing radiograph, (**C**) prepared cavity after selective caries excavation, (**D**) finished composite restoration overlying Activa Bioactive liner.

**Figure 3:**
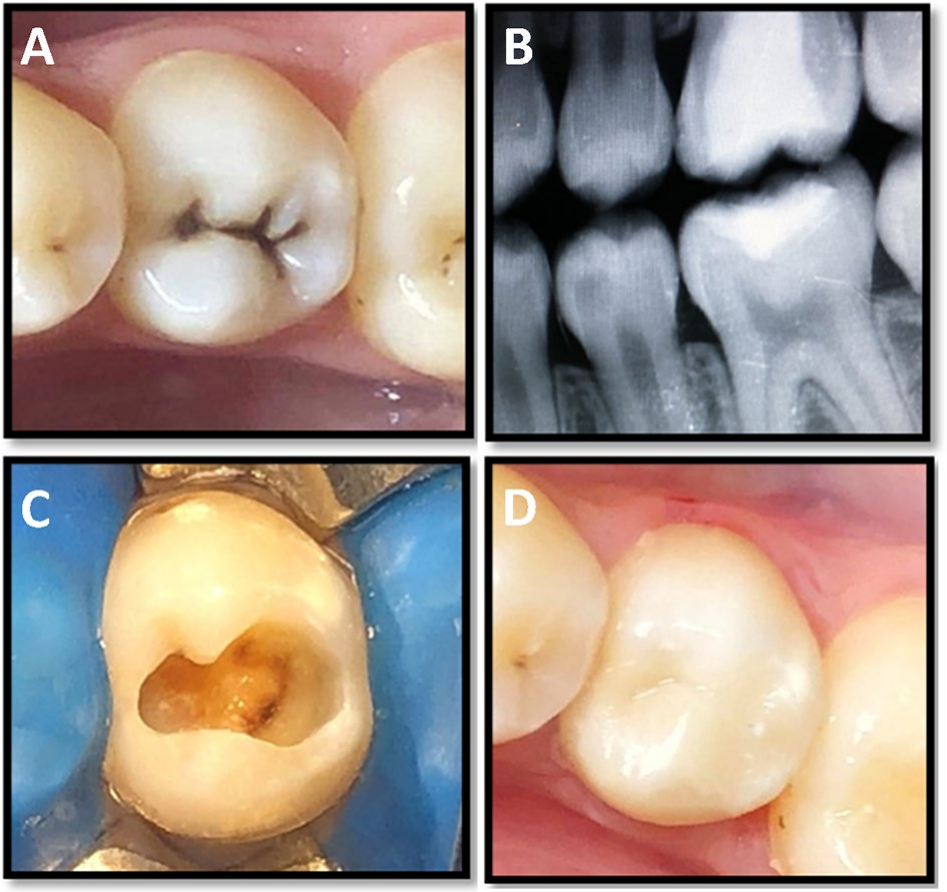
A representative of mandibular second premolar tooth showing; (**A**) preoperative photograph, (**B**) preoperative digital bitewing radiograph, (**C**) prepared cavity after selective caries excavation, (**D**) finished composite restoration overlying Riva Light Cure liner.

Following lining material placement, selective enamel etching was performed by application of 34% phosphoric acid gel (Scotchbond Etchant, 3M Oral Care, St Paul, MN, USA) on the enamel margins of prepared cavities for 15 s. The preparation was then thoroughly rinsed by water for 10 s and gently air dried. The universal bonding agent (Single Bond Universal, 3M Oral Care, St. Paul, MN, USA) was rubbed on the etched enamel and dentin surface by microbrush for 20 s. To evaporate the solvent, the bonding agent was gently air dried for 5 s followed by light curing for 10 s. Filtek Z350XT nanofilled resin composite (3M Oral Care, St. Paul, MN, USA) was applied incrementally to the prepared cavities using gold plated instrument. Each increment (2 mm thickness) was obliquely placed and light cured for 20 s. For restoring Class II cavities, a sectional coated metal matrix band with a ring and plastic wedge (Palodent, Dentsply DeTrey, konstanz, Germany) was used^26^. The occlusion was checked using articulating paper (Swedish Dental Supplies Ab, Akarp, Sweden) for establishing proper occlusal morphology and contact. The restorations were finished by using a high-speed diamond finishing instruments (4092.314, Komet) under copious air–water cooling. Polishing was performed using composite polishing kit (Shofu Inc, Kyoto, Japan) and Sof-lex discs (3M Oral Care, ST. Paul, MN, USA). The clinical procedures are presented in Figs. 2 and 3**.**

### Clinical evaluation

All restorations were evaluated clinically at baseline (1 week after the placement of restorations) and after 6 months, 1, 2 and 3 years according to FDI criteria. The clinical outcomes were evaluated according to the criteria in the following scores: (clinically very good, clinically good, clinically sufficient/satisfactory, clinically unsatisfactory, and clinically poor)^22,23^.

The evaluation process was carried out by two independent calibrated examiners who were blinded to the tested lining materials and did not contribute in the restoration process. The inter-examiner agreement was measured using Cohen’s Kappa coefficient. Disagreement occurred throughout evaluation process was solved by reevaluating the restorations by both examiners and obtaining a consensus before the patients left^31^. At the beginning of the assessment, each patient was instructed to brush his/her teeth for 3 min in order to remove dental plaque and food debris. The occlusal surface of the restoration was then dried with gentle air stream. A magnifying loupe was used as an additional diagnostic tool to assess in the visual examination of the restorations beside the standard illumination of the dental unit to ensure the validity of results^32^. All the FDI criteria were used for evaluating the lining materials and restorations. All the evaluation methods are illustrated in Table 2.

**Table 2:** Different methods used for clinical evaluation.

Criteria	Evaluation method
1. Surface luster	The operator light was switched off and the evaluation was performed at a distance of 60–100 cm (speaking distance)
2. Surface and marginal staining	Clinical inspection using dental mirror and operating light
3. Color match and translucency	The operator light was switched off and the evaluation was performed at a distance of 60–100 cm
4. Esthetic anatomical form	The operator light was switched off and the evaluation was performed at a distance of 60–100 cm
5. Fracture of material and retention	A magnifying aid (loupe 4.5 ×) was used for evaluation
6. Marginal adaptation	A magnifying aid (loupe 4.5 x) was used for evaluation Two special probes with different blunt tips (150 and 250 μm)
7. Occlusal contour and wear (qualitative)	Photodocumentation (baseline and follow-up images) for the occlusal surface of each restored tooth with and without contact areas marked with articulating paper
8. Approximal Anatomical form (contact point and contour)	The same type of waxed dental floss was used for evaluation at baseline and at all recalls
9. Radiographic examination	Periapical and bitewing radiographs
10. Patient’s view	A structured interview with the patient on his/her satisfaction/dissatisfaction with the restoration using Visual analogue scale (VAS)
11. Postoperative (hyper) sensitivity and tooth vitality	Intensity was assessed with VAS Postoperative sensitivity was evaluated by blowing a stream of compressed air for 3 s at a distance of 2–3 cm from the restoration Vitality was tested with application of cold (dry ice) and compared the reaction with the adjacent vital teeth
12. Recurrence of caries (CAR), erosion, abfraction	Diagnosis of caries was carried out according to ICDAS using loupe, mirror and the same two special probes
13. Tooth integrity (enamel cracks, tooth fractures)	Evaluation was performed using loupe with the help of the blunt probes
14. Periodontal Response	Periodontal probe was used to compare the reaction of the gingival tissues of the restored tooth and an unrestored tooth in the same patient based on the (PBI) Papillary Bleeding Index
15. Adjacent mucosa	Broad clinical inspection of the mucosa in the oral cavity
16. Oral and general Health	Broad clinical inspection of the whole oral cavity and also the medical status and history of the patient (systemic diseases, allergies or medications)

### Statistical analysis

The study outcomes were tabularized, coded, and analyzed using Statistical Software Package Program (IBM-SPSS, V.26, Armonk, NY, USA). The normality of data was checked by Kolmogorov–Smirnov test. Since the data did not follow normal distribution pattern, the descriptive statistics were exhibited in the form of median, minimum, and maximum range. Non-parametric statistical procedures were utilized to test significance of difference for each criterion at a significance level of p < 0.05. Mann–whitney U test was used to statistically compare between the data of the two tested groups for every criterion in each follow-up period. Moreover, Friedman test was used to statistically compare between the data of the same group for every criterion throughout the different follow-up periods.

### Ethical approval

The current study was registered on clinical trials with a unique identification number (I.D. NCT05470959). Ethical approval was obtained before the start of the study. The study was approved by the Dental Research Ethics Committee of Mansoura University (approval no. M19091019). The procedures used in this study adhere to the tenets of Helsinki Declaration.

### Informed consent

All participants gave their informed consent prior to their inclusion in the study.

## Results

The patient’s recall rate was 100% at baseline and 95% at the remaining follow-up evaluations. The reason of the patient’s dropout was travelling abroad during the study. The demographic data and clinical characteristics of each group are presented in Table 3. The Cohen’s Kappa statistics showed strong inter-examiner agreement (Kappa = 0.90), and no statistically significant difference was observed in patients’ answers (p > 0.05). The results of the current clinical trial are illustrated in Tables 4 and 5.

**Table 3:** Demographic data and clinical characteristics of study subjects per treatment group.

Group	Subjects	Age	Male/female	Premolar/molar	Maxillary/mandiblar	Class I/class II	ICDAS 5/ICDAS 6
Activa Bioactive	20	20–35	11/9	8/12	7/13	5/15	18/2
Riva Light Cure	20	20–35	11/9	10/10	9/11	7/13	19/1

**Table 4:** Results of FDI criteria scores of the tested groups during the 3-year follow-up periods.

A. Esthetic properties	Score	Activa bioactive					Riva light cure				
		Baseline	6 months	1 year	2 years	3 years	Baseline	6 months	1 year	2 years	3 years
1. Surface luster	1	20	19	18	18	18	20	19	19	18	18
	2	0	0	1	1	1	0	0	0	1	1
	3	0	0	0	0	1	0	0	0	0	0
	4	0	0	0	0	0	0	0	0	0	0
	5	0	0	0	0	0	0	0	0	0	0
2. Surface and marginal staining	1	20	19	19	19	17	20	19	19	18	18
	2	0	0	0	0	2	0	0	0	1	1
	3	0	0	0	0	0	0	0	0	0	0
	4	0	0	0	0	0	0	0	0	0	0
	5	0	0	0	0	0	0	0	0	0	0
3. Color match and translucency	1	20	19	19	19	19	20	18	18	18	18
	2	0	0	0	0	0	0	1	1	1	1
	3	0	0	0	0	0	0	0	0	0	0
	4	0	0	0	0	0	0	0	0	0	0
	5	0	0	0	0	0	0	0	0	0	0
4. Esthetic anatomical form	1	20	19	19	19	19	20	19	19	19	19
	2	0	0	0	0	0	0	0	0	0	0
	3	0	0	0	0	0	0	0	0	0	0
	4	0	0	0	0	0	0	0	0	0	0
	5	0	0	0	0	0	0	0	0	0	0

B. Functional properties	Score	Activa bioactive					Riva light cure				
		Baseline	6 months	1 year	2 years	3 years	Baseline	6 months	1 year	2 years	3 years
5. Fracture of material and retention	1	20	19	19	19	19	20	19	19	19	19
	2	0	0	0	0	0	0	0	0	0	0
	3	0	0	0	0	0	0	0	0	0	0
	4	0	0	0	0	0	0	0	0	0	0
	5	0	0	0	0	0	0	0	0	0	0
6. Marginal adaptation	1	20	19	19	19	17	20	19	19	18	18
	2	0	0	0	0	2	0	0	0	1	1
	3	0	0	0	0	0	0	0	0	0	0
	4	0	0	0	0	0	0	0	0	0	0
	5	0	0	0	0	0	0	0	0	0	0
7. Occlusal contour and wear	1	20	19	19	19	19	20	19	19	19	19
	2	0	0	0	0	0	0	0	0	0	0
	3	0	0	0	0	0	0	0	0	0	0
	4	0	0	0	0	0	0	0	0	0	0
	5	0	0	0	0	0	0	0	0	0	0
9. Radiographic examination	1	20	19	19	19	19	20	19	19	19	19
	2	0	0	0	0	0	0	0	0	0	0
	3	0	0	0	0	0	0	0	0	0	0
	4	0	0	0	0	0	0	0	0	0	0
	5	0	0	0	0	0	0	0	0	0	0
10. Patient’s view	1	20	18	18	18	18	20	18	18	18	18
	2	0	1	1	1	1	0	1	1	1	1
	3	0	0	0	0	0	0	0	0	0	0
	4	0	0	0	0	0	0	0	0	0	0
	5	0	0	0	0	0	0	0	0	0	0

C. Biological properties	Score	Activa bioactive					Riva light cure				
		Baseline	6 months	1 year	2 years	3 years	Baseline	6 months	1 year	2 years	3 years
11. Post-operative (hyper-) sensitivity and tooth vitality	1	20	18	19	19	19	20	17	18	18	18
	2	0	1	0	0	0	0	2	1	1	1
	3	0	0	0	0	0	0	0	0	0	0
	4	0	0	0	0	0	0	0	0	0	0
	5	0	0	0	0	0	0	0	0	0	0
12. Recurrence of caries (CAR), erosion, abfraction	1	20	19	19	19	19	20	19	19	19	19
	2	0	0	0	0	0	0	0	0	0	0
	3	0	0	0	0	0	0	0	0	0	0
	4	0	0	0	0	0	0	0	0	0	0
	5	0	0	0	0	0	0	0	0	0	0
13. Tooth integrity (enamel cracks, tooth fractures)	1	20	19	19	19	19	20	19	19	19	19
	2	0	0	0	0	0	0	0	0	0	0
	3	0	0	0	0	0	0	0	0	0	0
	4	0	0	0	0	0	0	0	0	0	0
	5	0	0	0	0	0	0	0	0	0	0
16. Oral and general health	1	20	19	19	19	19	20	19	19	19	19
	2	0	0	0	0	0	0	0	0	0	0
	3	0	0	0	0	0	0	0	0	0	0
	4	0	0	0	0	0	0	0	0	0	0
	5	0	0	0	0	0	0	0	0	0	0

**Table 5:** Results of FDI criteria scores of tested groups (Class II restorations only) during the 3-year follow-up periods.

B. Functional properties	Score	Activa bioactive					Riva light cure				
		Baseline	6 months	1 year	2 years	3 years	Baseline	6 months	1 year	2 years	3 years
8. Approximal anatomical form (contact point and contour)	1	14	14	14	14	14	12	12	12	12	12
	2	1	1	1	1	1	1	1	1	1	1
	3	0	0	0	0	0	0	0	0	0	0
	4	0	0	0	0	0	0	0	0	0	0
	5	0	0	0	0	0	0	0	0	0	0

C. Biological properties	Score	Activa bioactive					Riva light cure				
		Baseline	6 months	1 year	2 years	3 years	Baseline	6 months	1 year	2 years	3 years
14. Periodontal response (always compared to a reference tooth)	1	15	15	15	15	15	13	13	13	13	13
	2	0	0	0	0	0	0	0	0	0	0
	3	0	0	0	0	0	0	0	0	0	0
	4	0	0	0	0	0	0	0	0	0	0
	5	0	0	0	0	0	0	0	0	0	0
15. Adjacent mucosa	1	15	15	15	15	15	13	13	13	13	13
	2	0	0	0	0	0	0	0	0	0	0
	3	0	0	0	0	0	0	0	0	0	0
	4	0	0	0	0	0	0	0	0	0	0
	5	0	0	0	0	0	0	0	0	0	0

**Figure 4:**
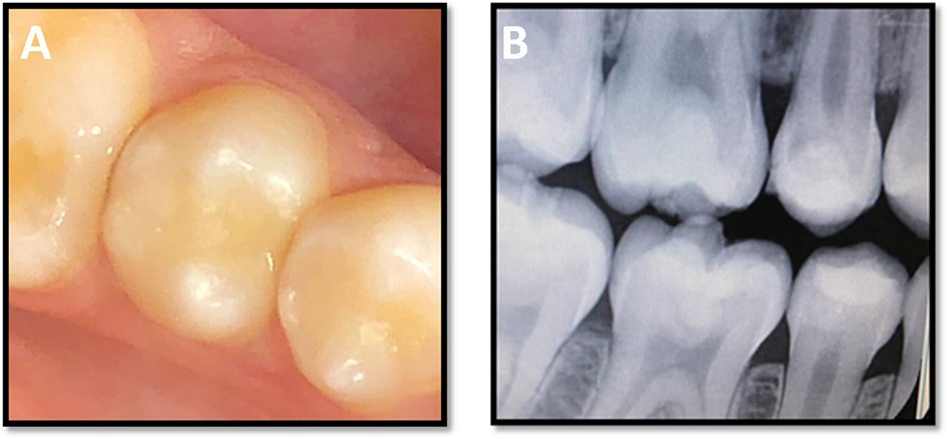
A representative of resin composite restoration lined with Activa Bioactive in mandibular second premolar tooth after 3 years showing: (**A**) clinical photograph, (**B**) digital bitewing radiograph.

**Figure 5:**
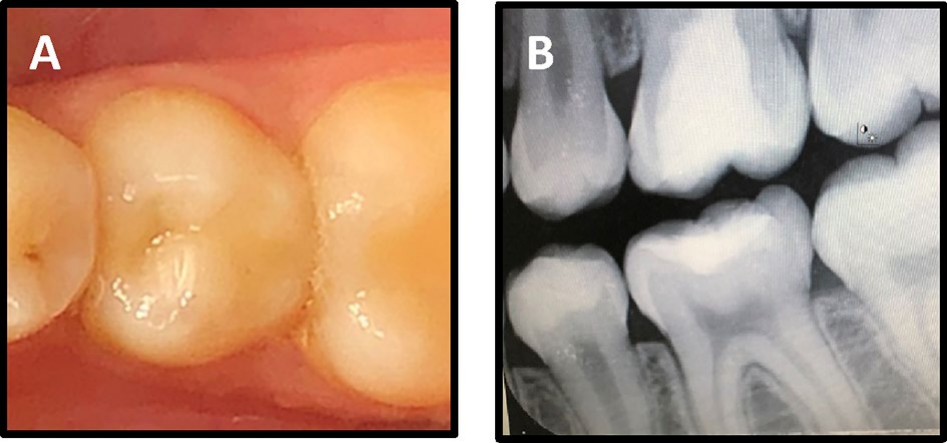
A representative of resin composite restoration lined with Riva Light Cure in mandibular second premolar tooth after 3 years showing: (**A**) clinical photograph, (**B**) digital bitewing radiograph.

**Figure 6:**
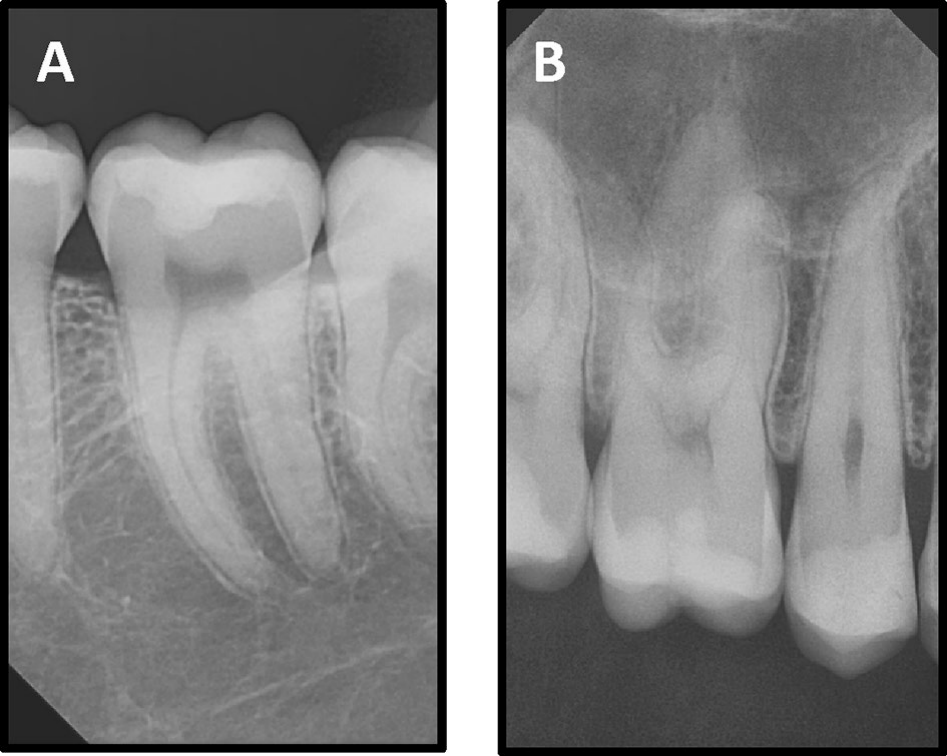
A representative periapical radiograph showing no pathological findings related to composite restorations lined with; (**A**) activa Bioactive, (**B**) riva light cure.

The success rate for both groups was 100% after 3 years (Figs. 4 and 5). The scores of clinical evaluations for the current study showed no statistically significant differences between the two tested groups at baseline and after 6 months, 1, 2, and 3 years (p > 0.05). Also, no significant changes over time were detected for both groups for any of the evaluation criteria (p > 0.05). Radiographic examination using digital periapical radiograph showed that there were no pathological changes related to any restoration lined neither with Activa Bioactive nor Riva Light Cure after 3 years (Fig. 6).

## Discussion

The current clinical trial investigated the 3-year performance of posterior resin composite restorations lined with Activa Bioactive and Riva Light Cure after selective excavation of deep carious lesions. The study results showed that there was no statistically significant difference between both groups by the end of 3-year follow-up. Therefore, the null hypothesis formulated at the beginning of this clinical trial was accepted.

Laboratory studies participate in initial assessment of dental materials providing valuable data regarding their potential performance. However, this does not precisely reveal their clinical performance due to the presence of variable parameters in oral cavity including hydrolytic attacks, pH variations, and temperature changes^33,34^. Therefore, clinical trials are usually considered the preferable method for evaluating the clinical performance and longevity of newly developed dental materials^25^. Split-mouth design was chosen for within-patient comparisons rather than between-patient comparisons aiming to expose all of the tested materials to the same oral environmental conditions^35^. The present clinical study was a double-blinded study in order to eliminate examiner or patient related bias^25^.

The removal of caries-infected dentin without affecting remineralizable dentin has been the ultimate goal of minimal invasive dentistry^30^. Polymer burs were used in the current study due to their self-limiting ability in caries excavation and hence, more tooth structure preservation^12^. This was confirmed by Freedman and Goldstep^36^ who reported that polymer burs were efficient in removing infected carious dentin selectively as their cutting edges blunted out when they became in contact with hard or affected dentin.

The 3-year clinical evaluation of esthetic properties did not show any significant differences for all resin composite restorations overlying both liners. This could be attributed to the presence of nanofillers with particle size below the wavelength of visible light leading to significant light absorption without light scattering and so, superior esthetic outcomes^37^. Also, nanofiller particles provide composites with high color stability, high translucency, high polish retention, low surface roughness, and low staining susceptibility^38^. This result is in agreement with Mahmoud et al.^39^ who reported that nanofilled composite restorations had excellent color match with the surrounding tooth structure in addition to the high surface luster after 2-year follow-up period. Another clinical trial performed by Dresch et al.^40^ stated that using nanofilled composite in posterior teeth showed high esthetic outcomes after 1 year of clinical examination.

Only three composite restorations suffered from a slight change in staining criterion after 3 years. This marginal staining occurring in the crevice between the restoration and the cavity wall could be related to pigment and stains absorption from dietary habits. The use of liners in this study did not have any significant influence on the esthetic outcomes, since they were used in a thin layer that could not be seen through^26^.

No composite restorations were fractured during the 3-year follow-up. This could be attributed to the combination of nanomer and nanocluster filler particles in nanofilled composite which decreases the interstitial spacing leading to high filler loading and enhanced physical and mechanical properties^41^. Moreover, the nanofillers might act as point that could prevent crack propagation and restoration fracture^42^. This result is in accordance with a previous study^43^ which showed that nanofilled resin composite had high flexural strength and excellent mechanical properties.

Resin composite restorations exhibited excellent marginal adaptation with no significant differences detected during the different recall periods. This could be related to the application of universal adhesive system which enhanced the bond durability owing to presence of functional monomer (10-MDP) that interacts chemically with hydroxyapatites. Selective enamel etching also increased the bond strength to enamel and resulted in long-lasting marginal seal^44^. The presence of remaining remineralizable dentin surrounded by sound dentin after selective caries excavation did not has a negative effect on the marginal integrity of composite restorations as confirmed by Scholz et al.^45^. A study conducted by Sauro et al.^46^ showed that ion-releasing lining materials had an important role on the longevity of resin composite restorations bonded by universal adhesive. All marginal gaps were detected using two special probes (150 and 250 μm) during the follow-up periods^22^. Only three restorations showed a slight change in marginal adaptation over time which could be resulted from the degradation of the resin/bond interface as a result of slow water hydrolysis.

Clinical evaluation of wear was performed to assist in understanding the restorative material behavior when submitted to the complex oral masticatory changes. Based on the findings of this clinical study, resin composite restorations did not show any significant alterations in occlusal contour during all of the recall visits. This could be attributed to the higher flexural strength and wear resistance of nanofilled resin composite as reported in previous study^42^. It has been suggested that wear of resin composite is dependent on filler loading, formulation of resin matrix, adhesion of fillers to the matrix and filler size^47^. This result was confirmed by Frankenberger et al.^48^ who reported that nanocomposites had satisfactory clinical performance regarding the wear rates over the 8-year observation period. In contrast, Yesil et al.^49^ reported that incorporation of nanofillers in resin composite materials did not significantly enhance wear resistance.

Digital bitewing and periapical radiographs were taken to aid in the detection of restoration gaps, secondary caries, apical pathological changes, and material loss that might be difficult to be detected clinically^22^. The results of this study showed no differences in radiographic outcomes between both tested groups during the 3-year evaluations. Moreover, no periapical changes related to any restoration were found. This could be attributed to bioactivity and fluoride release of the used cavity liners which enhanced the remineralization of caries-affected dentin and prevented caries progression preserving pulp vitality.

Each patient was asked about his/her opinion regarding esthetics, pain, function, hypersensitivity, and comfort during chewing. Most of patients were satisfied with their restorations, where no esthetic or functional problems were detected. However, only two patients reported minor criticism which could be related to the changes in restorations’ surface texture.

The clinical evaluation for postoperative hypersensitivity involved type of pain, duration, and stimulating factors. Also, the effect of the tested lining materials on pulp vitality response was evaluated by comparing the reaction of restored tooth to that of a vital unrestored tooth using cold ice^22^. According to outcomes of this clinical trial, there was no significant difference regarding postoperative hypersensitivity between both tested groups through the follow-up periods. This could be attributed to the active ionic resin matrix and shock absorbing rubberized fillers in Activa Bioactive liner which bond chemically to tooth structure and provide sealing against bacterial leakage and thus, reducing sensitivity. Moreover, Activa Bioactive has the ability to release and recharge calcium, phosphate, and fluoride ions provoking the mineral apatite formation and remineralization^50^. This was supported by Hirani et al.^51^ who reported that there was no postoperative hypersensitivity related to Activa Bioactive material. A previous report^52^ stated that Activa Bioactive had higher degree of biocompatibility and healing ability when compared to calcium silicate-based cement. Conversely, Van Dijken et al.^53^ reported that the postoperative hypersensitivity of Activa Bioactiva could be related the severe weakness of initial bond to the cavity walls which led to progressing deterioration of material adaptation. Furthermore, a clinical study performed by Weston et al.^54^ concluded that using RMGI liners prevented hypersensitivity and microleakage owing to the extended fluoride release and the remineralization effect. A previous study^38^ reported that RMGI materials had minimum sensitivity which could be explained by the durable bond to dentin, dimensional stability, and excellent adaptation with the tooth structure. On the contrary, Strober et al.^55^ reported that using RMGI liners did not decrease the clinically evaluated postoperative hypersensitivity.

The absence of recurrent and secondary caries over time could be related to the selection of well-motivated participants with good oral hygiene status in addition to the instructions given to all of them after the placement of the restorations. Moreover, the proper restorative techniques and the adequate marginal seal of the restorations prevented the bacterial penetration. The antibacterial and remineralizing ability of Activa Bioactive and Riva Light Cure liners had a crucial role in caries prevention, as well both lining materials showed high bond strength to dentin and resin composite which might prevent secondary caries and microleakage as reported by a previous laboratory study^50^. This finding was in agreement with a clinical trial^56^ which stated that both Activa Bioactive and RMGI scored 100% success after 1 year regarding the recurrence of decay. Conversely, Abou ElReash et al.^57^ reported that Activa Bioactive had weak antibacterial properties due to its resinous ingredients and scantily acidic nature.

Clinical investigation of tooth integrity was performed visually to detect tooth or enamel fracture, cracks, and chipping^23^. No tooth fracture was detected during the different recalls for both groups. This could be related to the minimal invasive cavity preparation which prevented the weakening of remaining tooth structure and maintained its integrity. In the present study, none of cases showed oral or general symptoms during the 3-year follow-up periods. This might be related to the exclusion of patients with adverse medical conditions and systemic diseases. Furthermore, all the materials used in this study were approved by the FDA.

The clinical evaluation of proximal contact and contour for Class II restorations was carried out by passing waxed dental floss through contact area to detect contact deficiency which might lead to food impaction, plaque accumulation, secondary caries, and patient discomfort^22,23^. The current study results indicated no significant differences in approximal anatomical form between both groups throughout the follow-up periods. This could be explained by utilization of the sectional matrix system and wedges that led to strong contact points and correct contours in Class II restorations. This was confirmed by De la Pena et al.^58^ who stated that using sectional matrix was the best way to accomplish strong contact point in Class II restorations. When compared baseline to the different recall periods, no gingival inflammation, plaque accumulation, or pockets were found related to any restoration. Also, all cases showed healthy mucosa adjacent to the restorations without any allergic reactions or periodontal problems. This could be related to the well-established contacts and contours which prevented the food impaction.

The outcomes of the current clinical trial are limited by small sample size and limited number of treated teeth in addition to the short follow-up period. It would be recommended to perform future long-term randomized clinical trials evaluating ion-releasing liners in large number of patients with high recall rates to test these materials for further clinical applications.

## Conclusion

Resin composite restorations showed acceptable clinical performance over 3 years either lined with bioactive ionic or resin-modified glass ionomer liners after selective caries excavation preserving pulp vitality.

## Data Availability

The datasets used and/or analyzed during the current study available from the corresponding author on reasonable request.

## References

[1] Schwendicke F, Göstemeyer G (2016). Understanding dentists' management of deep carious lesions in permanent teeth: A systematic review and meta-analysis. Implement. Sci..

[2] Veiga N, Figueiredo R, Correia P, Lopes P, Couto P, Fernandes GVO (2023). Methods of primary clinical prevention of dental caries in the adult patient: An integrative review. Healthcare (Basel).

[3] Schwendicke F, Walsh T, Lamont T, Al-Yaseen W, Bjorndal L, Clarkson JE, Lamont T, Levey C, Gostemeyer G, Santamaria RM, Ricketts D, Innes NPT (2021). Interventions for treating cavitated or dentin carious lesions. Cochrane Database Syst. Rev..

[4] Bjørndal L, Fransson H, Bruun G, Markvart M, Kjældgaard M, Näsman P, Hedenbjörk-Lager A, Dige I, Thordrup M (2017). Randomized clinical trials on deep carious lesions: 5-year follow-up. J. Dent. Res..

[5] Thompson V, Craig RG, Curro FA, Green WS, Ship JA (2008). Treatment of deep carious lesions by complete excavation or partial removal: A critical review. J. Am. Dent. Assoc..

[6] Bitello-Firmino L, Soares VK, Damé-Teixeira N, Parolo CCF, Maltz M (2018). Microbial load after selective and complete caries removal in permanent molars: A randomized clinical trial. Braz. Dent. J..

[7] Singhal DK, Acharya S, Thakur AS (2016). Microbiological analysis after complete or partial removal of carious dentin using two different techniques in primary teeth: A randomized clinical trial. Dent. Res. J..

[8] Asal MA, Abdellatif AM, Hammouda HE (2021). Clinical and microbiological assessment of carisolv and polymer bur for selective caries removal in primary molars. Int. J. Clin. Pediatr. Dent..

[9] Ferraz C, Freire AR, Mendonca JS, Fernandes CA, Cardona JC, Yamauti M (2015). Effectiveness of different mechanical methods on dentin caries removal: Micro-CT and digital image evaluation. Oper. Dent..

[10] Boston DW (2003). New device for selective dentin caries removal. Quintessence Int..

[11] De Almeida NA, Coutinho E, Cardoso MV, Lambrechts P, Van Meerbeek B (2011). Current concepts and techniques for caries excavation and adhesion to residual dentin. J. Adhes. Dent..

[12] Toledano M, Ghinea R, Cardona JC, Cabello I, Yamauti M, Perez MM, Osorio R (2013). Digital image analysis method to assess the performance of conventional and self-limiting concepts in dentine caries removal. J. Dent..

[13] Meller C, Welk A, Zeligowski T, Splieth C (2007). Comparison of dentin caries excavation with polymer and conventional tungsten carbide burs. Quintessence Int..

[14] Singh S, Mittal S, Tewari S (2019). Effect of different liners on pulpal outcome after partial caries removal: A preliminary 12 months randomised controlled trial. Caries Res..

[15] Qureshi A, Nandakumar ES, Sambashivarao P (2014). Recent advances in pulp capping materials: An overview. J. Clin. Diagn. Res..

[16] Schwendicke F, Meyer-Lueckel H, Dörfer C, Paris S (2013). Failure of incompletely excavated teeth—a systematic review. J. Dent..

[17] Stafuzza TC, Vitor LLR, Rios D, Cruvinel T, Loureço Neto N, Sakai VT, Machado MAAM, Oliveira TM (2019). A randomized clinical trial of cavity liners after selective caries removal: One-year follow-up. J. Appl. Oral Sci..

[18] Kunert M, Lukomska-Szymanska M (2020). Bio-inductive materials in direct and dental repair material: A resin-modified glass ionomer bioactive ionic resin-based composite indirect pulp capping—a review article. Materials.

[19] Almuhaiza M (2016). Glass-ionomer cements in restorative dentistry: A critical appraisal. J. Contemp. Dent. Pract..

[20] Croll TP, Berg JH, Donly KJ (2015). Dental repair material: A resin-modified glass-ionomer bioactive ionic resin-based composite. Compend. Contin. Educ. Dent..

[21] İnci MA, Korkut E (2022). Is bioactive glass an effective agent in pulp-capping treatments?: A randomized controlled clinical trial with one-year follow-up. J. Contemp. Dent. Pract..

[22] Hickel R, Peschke A, Tyas M, Mjör I, Bayne S, Peters M, Hiller KA, Randall R, Vanherle G, Heintze SD (2010). FDI world dental federation: Clinical criteria for the evaluation of direct and indirect restorations-update and clinical examples. Clin. Oral Investig..

[23] Hickel R, Roulet JF, Bayne S, Heintze SD, Mjör IA, Peters M, Rousson V, Randall R, Schmalz G, Tyas M, Vanherle G (2007). Recommendations for conducting controlled clinical studies of dental restorative materials. Clin. Oral Investig..

[24] Schulz KF, Altman DG, Moher D, CONSORT Group (2010). CONSORT 2010 Statement: Updated guidelines for reporting parallel group randomised trials. BMC Med..

[25] Bhadra D, Shah NC, Rao AS, Dedania MS, Bajpai N (2019). A 1-year comparative evaluation of clinical performance of nanohybrid composite with Activa™ bioactive composite in Class II carious lesion: A randomized control study. J. Conserv. Dent..

[26] Torres CRG, Mailart MC, Rocha RS, Sellan PLB, Contreras SCM, Di Nicoló R, Borges AB (2020). The influence of a liner on deep bulk-fill restorations: Randomized clinical trial. J. Dent..

[27] Young DA, Nový BB, Zeller GG, Hale R, Hart TC, Truelove EL, American Dental Association Council on Scientific Affairs (2015). The American Dental Association Caries Classification System for clinical practice: A report of the American Dental Association Council on Scientific Affairs. J. Am. Dent. Assoc..

[28] Urzúa I, Cabello R, Marín P, Ruiz B, Jazanovich D, Mautz C, Lira M, Sánchez J, Rodríguez G, Osorio S, Ortiz ME (2019). Detection of approximal caries lesions in adults: A cross-sectional study. Oper. Dent..

[29] Wafaie RA, Ibrahim Ali A, El-Negoly SAE, Mahmoud SH (2023). Five-year randomized clinical trial to evaluate the clinical performance of high-viscosity glass ionomer restorative systems in small class II restorations. J. Esthet. Restor. Dent..

[30] Prabhakar A, Kiran NK (2009). Clinical evaluation of polyamide polymer burs for selective carious dentin removal. J. Contemp. Dent. Pract..

[31] Mahmoud SH, Ali AK, Hegazi HA (2014). A three-year prospective randomized study of silorane-and methacrylate-based composite restorative systems in class II restorations. J. Adhes. Dent..

[32] Hickel R, Mesinger S, Opdam N, Loomans B, Frankenberger R, Cadenaro M, Burgess J, Peschke A, Heintze SD, Kühnisch J (2023). Revised FDI criteria for evaluating direct and indirect dental restorations-recommendations for its clinical use, interpretation, and reporting. Clin. Oral Investig..

[33] Arhun N, Celik C, Yamanel K (2010). Clinical evaluation of resin-based composites in posterior restorations: Two-year results. Oper. Dent..

[34] Balkaya H, Arslan S, Pala K (2019). A randomized, prospective clinical study evaluating effectiveness of a bulk-fill composite resin, a conventional composite resin and a reinforced glass ionomer in class II cavities: One-year results. J. Appl. Oral Sci..

[35] Zanatta RF, Da Silva TM, Esper MALR, Bresciani E, Caneppele TMF, Goncalves SEP (2017). Guidelines for conducting split-mouth clinical studies in restorative dentistry. Braz. Dent. Sci..

[36] Freedman G, Goldstep F (2003). Polymer preparation instruments. New paradigm in selective dentin removal. Dent. Today.

[37] Rocha Maia R, Oliveira D, D'Antonio T, Qian F, Skiff F (2018). Comparison of light-transmittance in dental tissues and dental composite restorations using incremental layering build-up with varying enamel resin layer thickness. Restor. Dent. Endod..

[38] Mushtaq U, Mushtaq F, Thakur D, Rathee K, Poonia N, Khullar S (2021). Comparative evaluation of postoperative sensitivity following restoration of class I lesions with different restorative materials: An in vivo study. J. Contemp. Dent. Pract..

[39] Mahmoud SH, El-Embaby AE, AbdAllah AM, Hamama HH (2008). Two year clinical evaluation of ormocer, nanohybrid and nanofillcomposite restorative systems in posterior teeth. J. Adhes. Dent..

[40] Dresch W, Volpato S, Gomes JC, Ribeiro NR, Reis A, Loguercio AD (2006). Clinical evaluation of a nanofilled composite in posterior teeth: 12-month results. Oper. Dent..

[41] Hamouda IM, Abd Elkader H (2012). Evaluation the mechanical properties of nanofilled composite resin restorative material. J. Biomater. Nanobiotechnol..

[42] Alzraikat H, Burrow M, Maghaireh G, Taha N (2018). Nanofilled resin composite properties and clinical performance: A review. Oper. Dent..

[43] Beun S, Glorieux T, Devaux J, Vreven J, Leloup G (2007). Characterization of nanofilled compared to universal and microfilled composites. Dent. Mater..

[44] Hanabusa M, Mine A, Kuboki T, Momoi Y, Van Ende A, Van Meerbeek B, De Munck J (2012). Bonding effectiveness of a new multi-mode adhesive to enamel and dentine. J. Dent..

[45] Scholz KJ, Hinderberger M, Widbiller M, Federlin M, Hiller KA, Buchalla W (2020). Influence of selective caries excavation on marginal penetration of class II composite restorations in vitro. Eur. J. Oral Sci..

[46] Sauro S, Makeeva I, Faus-Matoses V, Foschi F, Giovarruscio M, Maciel Pires P, Martins Moura ME, Almeida Neves A, Faus-Llácer V (2023). Effects of ions-releasing restorative materials on the dentine bonding longevity of modern universal adhesives after load-cycle and prolonged artificial saliva aging. Mater. (Basel).

[47] Turssi CP, De Moraes PB, Serra MC (2003). Wear of dental resin composites: Insights into underlying processes and assessment methods—a review. J. Biomed. Mater. Res. B Appl. Biomater..

[48] Frankenberger R, Reinelt C, Kramer N (2014). Nanohybrid vs. fine hybrid composite in extended class II cavities: 8-year results. Clin. Oral Investig..

[49] Yesil ZD, Alapati S, Johnston W, Seghi RR (2008). Evaluation of the wear resistance of new nanocomposite resin restorative materials. J. Prosthet. Dent..

[50] Ahmed B, Hamama HH, Mahmoud SH (2023). Microshear bond strength of bioactive materials to dentin and resin composite. Eur. J. Dent..

[51] Hirani RT, Batra R, Kapoor S (2018). Comparative evaluation of postoperative sensitivity in bulkfill restoratives: A randomized trial. J. Int. Soc. Prev. Commun. Dent..

[52] Abou ElReash A, Hamama H, Abdo W, Wu Q, Zaen El-Din A, Xiaoli X (2019). Biocompatibility of new bioactive resin composite versus calcium silicate cements: An animal study. BMC Oral Health.

[53] Van Dijken JWV, Pallesen U, Benetti A (2019). A randomized controlled evaluation of posterior resin restorations of an altered resin modified glass-ionomer cement with claimed bioactivity. Dent. Mater..

[54] Weston J (2015). Use of a resin-modified glass-ionomer (RMGI) liner in conservative direct treatment of deep caries. Compend. Contin. Educ. Dent..

[55] Strober B, Veitz-Keenan A, Barna JA, Matthews AG, Vena D, Craig RG, Curro FA, Thompson VP (2013). Effectiveness of a resin-modified glass ionomer liner in reducing hypersensitivity in posterior restorations: A study from the practitioners engaged in applied research and learning network. J. Am. Dent. Assoc..

[56] Eissa MM, Akah M, Yousry MM, Hamza H, Hassanein H, Pameijer CH (2021). Clinical performance of a bioactive restorative material vs a glass hybrid restorative in posterior restorations in high-risk caries patients. World J. Dent..

[57] Abou ElReash A, Hamama H, Eldars W, Lingwei G, Zaen El-Din AM, Xiaoli X (2019). Antimicrobial activity and pH measurement of calcium silicate cements versus new bioactive resin composite restorative material. BMC Oral Health.

[58] De la Pena VA, Garcia RP, Garcia RP (2016). Sectional matrix: Step by step directions for their clinical use. Br. Dent. J..

